# Lifetime ovulatory years and risk of epithelial ovarian cancer: a multinational pooled analysis

**DOI:** 10.1093/jnci/djad011

**Published:** 2023-05-08

**Authors:** Zhuxuan Fu, Maria Mori Brooks, Sarah Irvin, Susan Jordan, Katja K.H. Aben, Hoda Anton-Culver, Elisa V. Bandera, Matthias W. Beckmann, Andrew Berchuck, Angela Brooks-Wilson, Jenny Chang-Claude, Linda S. Cook, Daniel W. Cramer, Kara L. Cushing-Haugen, Jennifer A. Doherty, Arif B. Ekici, Peter A. Fasching, Renée T. Fortner, Simon A. Gayther, Aleksandra Gentry-Maharaj, Graham G. Giles, Ellen L. Goode, Marc T. Goodman, AOCS Group, Holly R. Harris, Alexander Hein, Rudolf Kaaks, Lambertus A. Kiemeney, Martin Köbel, Joanne Kotsopoulos, Joanne Kotsopoulos, Nhu D. Le, Alice W. Lee, Keitaro Matsuo, Valerie McGuire, John R. McLaughlin, Usha Menon, Roger L. Milne, Kirsten B. Moysich, Celeste Leigh Pearce, Malcolm C. Pike, Bo Qin, Susan J. Ramus, Marjorie J. Riggan, Joseph H. Rothstein, Joellen M. Schildkraut, Weiva Sieh, Rebecca Sutphen, Kathryn L. Terry, Pamela J. Thompson, Linda Titus, Anne M. van Altena, Emily White, Alice S. Whittemore, Anna H. Wu, Wei Zheng, Argyrios Ziogas, Sarah E. Taylor, Lu Tang, Thomas Songer, Nicolas Wentzensen, Penelope M. Webb, Harvey A. Risch, Francesmary Modugno

**Affiliations:** 1 Department of Epidemiology, University of Pittsburgh Graduate School of Public Health, Pittsburgh, PA, USA; 2 Department of Biostatistics, University of Pittsburgh Graduate School of Public Health, Pittsburgh, PA, USA; 3 Division of Cancer Epidemiology and Genetics, National Cancer Institute, Rockville, MD, USA; 4 The School of Public Health, The University of Queensland, Brisbane, Queensland; 5 Radboud Institute for Health Sciences, Radboud University Medical Center, Nijmegen, the Netherlands; 6 Netherlands Comprehensive Cancer Organisation, Utrecht, the Netherlands; 7 Department of Medicine, Genetic Epidemiology Research Institute, University of California Irvine, Irvine, CA, USA; 8 Cancer Prevention and Control Program, Rutgers Cancer Institute of New Jersey, New Brunswick, NJ, USA; 9 Department of Gynecology and Obstetrics, Comprehensive Cancer Center Erlangen-EMN, Friedrich-Alexander University Erlangen-Nuremberg, University Hospital Erlangen, Erlangen, Germany; 10 Department of Obstetrics and Gynecology, Division of Gynecologic Oncology, Duke University Medical Center, Durham, NC, USA; 11 Canada’s Michael Smith Genome Sciences Centre, BC Cancer, Vancouver, BC, Canada; 12 Division of Cancer Epidemiology, German Cancer Research Center (DKFZ), Heidelberg, Germany; 13 Cancer Epidemiology Group, University Cancer Center Hamburg (UCCH), University Medical Center Hamburg-Eppendorf, Hamburg, Germany; 14 Epidemiology, School of Public Health, University of Colorado, Aurora, CO, USA; 15 Community Health Sciences, University of Calgary, Calgary, AB, Canada; 16 Obstetrics and Gynecology Epidemiology Center, Department of Obstetrics and Gyneoclogy, Brigham and Women’s Hospital and Harvard Medical School, Boston, MA, USA; 17 Department of Epidemiology, Harvard T.H. Chan School of Public Health, Boston, MA, USA; 18 Program in Epidemiology, Division of Public Health Sciences, Fred Hutchinson Cancer Research Center, Seattle, WA, USA; 19 Huntsman Cancer Institute, Department of Population Health Sciences, University of Utah, Salt Lake City, UT, USA; 20 Institute of Human Genetics, Comprehensive Cancer Center Erlangen-EMN, University Hospital Erlangen, Friedrich-Alexander University Erlangen-Nuremberg (FAU), Erlangen, Germany; 21 Center for Bioinformatics and Functional Genomics and the Cedars Sinai Genomics Core, Cedars-Sinai Medical Center, Los Angeles, CA, USA; 22 MRC Clinical Trials Unit, Institute of Clinical Trials & Methodology, University College London, London, UK; 23 Cancer Epidemiology Division, Cancer Council Victoria, Melbourne, Victoria, Australia; 24 Centre for Epidemiology and Biostatistics, Melbourne School of Population and Global Health, The University of Melbourne, Melbourne, Victoria, Australia; 25 Precision Medicine, School of Clinical Sciences at Monash Health, Monash University, Clayton, Victoria, Australia; 26 Department of Quantitative Health Sciences, Division of Epidemiology, Mayo Clinic, Rochester, MN, USA; 27 Cancer Prevention and Control Program, Cedars-Sinai Cancer, Cedars-Sinai Medical Center, Los Angeles, CA, USA; 28 Cancer Genetics Laboratory, Research Division, Peter MacCallum Cancer Center, Melbourne, Victoria, Australia; 29 Centre for Cancer Research, The Westmead Institute for Medical Research, Sydney, New South Wales, Australia; 30 Department of Epidemiology, University of Washington, Seattle, WA, USA; 31 Department of Pathology and Laboratory Medicine, University of Calgary, Foothills Medical Center, Calgary, AB, Canada; 32 Women’s College Research Institute, Women’s College Hospital, University of Toronto, Toronto, Ontario, Canada; 33 Dalla Lana School of Public Health, University of Toronto, Toronto, Ontario, Canada; 34 Cancer Control Research, BC Cancer, Vancouver, BC, Canada; 35 Department of Health Science, California State University, Fullerton, Fullerton, CA, USA; 36 Division of Cancer Epidemiology and Prevention, Aichi Cancer Center Research Institute, Nagoya, Japan; 37 Division of Cancer Epidemiology, Nagoya University Graduate School of Medicine, Nagoya, Japan; 38 Department of Epidemiology and Population Health, Stanford University School of Medicine, Stanford, CA, USA; 39 Public Health Ontario, Samuel Lunenfeld Research Institute, Toronto, Ontario, Canada; 40 Department of Cancer Prevention and Control, Roswell Park Cancer Institute, Buffalo, NY, USA; 41 Department of Epidemiology, University of Michigan School of Public Health, Ann Arbor, MI, USA; 42 Department of Epidemiology and Biostatistics, Memorial Sloan-Kettering Cancer Center, New York, NY, USA; 43 Department of Population Health and Public Health Sciences, Keck School of Medicine, University of Southern California Norris Comprehensive Cancer Center, Los Angeles, CA, USA; 44 School of Clinical Medicine, UNSW Medicine and Health, University of NSW Sydney, Sydney, New South Wales, Australia; 45 Adult Cancer Program, Lowy Cancer Research Centre, University of NSW Sydney, Sydney, New South Wales, Australia; 46 Department of Genetics and Genomic Sciences, Icahn School of Medicine at Mount Sinai, New York, NY, USA; 47 Department of Population Health Science and Policy, Icahn School of Medicine at Mount Sinai, New York, NY, USA; 48 Department of Epidemiology, Rollins School of Public Health, Emory University, Atlanta, GA, USA; 49 Epidemiology Center, College of Medicine, University of South Florida, Tampa, FL, USA; 50 Samuel Oschin Comprehensive Cancer Institute, Cancer Prevention and Genetics Program, Cedars-Sinai Medical Center, Los Angeles, CA, USA; 51 Muskie School of Public Policy, Public Health, Portland, ME, USA; 52 Department of Obstetrics and Gynaecology, Radboud University Medical Center, Nijmegen, the Netherlands; 53 Fred Hutchinson Cancer Research Center, Seattle, WA, USA; 54 Department of Biomedical Data Science, Stanford University School of Medicine, Stanford, CA, USA; 55 Division of Epidemiology, Department of Medicine, Vanderbilt Epidemiology Center, Vanderbilt-Ingram Cancer Center, Vanderbilt University School of Medicine, Nashville, TN, USA; 56 Division of Gynecologic Oncology, Department of Obstetrics, Gynecology and Reproductive Sciences, University of Pittsburgh School of Medicine, Pittsburgh, PA, USA; 57 Division of Cancer Epidemiology and Genetics, National Cancer Institute, Bethesda, MD, USA; 58 Chronic Disease Epidemiology, Yale School of Public Health, New Haven, CT, USA; 59 Women’s Cancer Research Center, Magee-Womens Research Institute and Hillman Cancer Center, Pittsburgh, PA, USA

**Keywords:** epithelial ovarian cancer, lifetime ovulation years, case-control study, incessant ovulation, pooled analysis, OCAC

## Abstract

**Background:**

The role of ovulation in epithelial ovarian cancer (EOC) is supported by the consistent protective effects of parity and oral contraceptive (OC) use. Whether these factors protect through anovulation alone remains unclear. We explored the association between lifetime ovulatory years (LOY) and EOC.

**Methods:**

LOY was calculated using 12 algorithms. Odds ratios (ORs) and 95% confidence intervals (CIs) estimated the association between LOY or LOY components and EOC among 26,204 controls and 21,267 cases from 25 studies. To assess whether LOY components act through ovulation suppression alone, we compared beta coefficients obtained from regression models to expected estimates assuming one year of ovulation suppression has the same effect regardless of source.

**Results:**

LOY was associated with increased EOC risk (ORs per year increase: 1.014 (95%CI 1.009-1.020) to 1.044 (95%CI 1.041-1.048)). Individual LOY components, except age at menarche, also associated with EOC. The estimated model coefficient for OC use and pregnancies were 4.45 times and 12-15 fold greater than expected, respectively. LOY was associated with high-grade serous (HGSOC), low-grade serous (LGSOC), endometrioid, and clear cell histotypes (ORs per year increase: 1.054, 1.040, 1.065, and 1.098, respectively), but not mucinous tumors. Estimated coefficients of LOY components were close to expected estimates for HGSOC but larger than expected for LGSOC, endometrioid, and clear cell histotypes.

**Conclusions:**

LOY is positively associated with non-mucinous EOC. Differences between estimated and expected model coefficients for LOY components suggest factors beyond ovulation underlie the associations between LOY components and EOC in general and for non-HGSOC.

Epithelial ovarian cancer (EOC) is the most lethal gynecologic malignancy. The consistent protective effects of oral contraceptives (OC),^1–3^ bearing children,^3,4^ and breastfeeding,^5^ which all suppress ovulation, suggest that ovulation may play a key role in disease origin.^6^ In support of this hypothesis, lifetime ovulatory years (LOY) have been associated with increased EOC risk.^2,7–14^ However, differences in how studies define LOY and categorize exposure make it challenging to quantify the LOY-EOC relationship.^15^ Moreover, it remains unclear whether the mechanism whereby LOY components exert their impacts is through ovulation suppression alone or other means.^7^

While EOC is considered a set of diseases defined by histologic subtypes (“histotypes”), the relationship between LOY and EOC histotypes remains understudied. Although LOY might be associated with specific EOC subtypes,^2,10–14^ no individual study has had a large enough sample size to undertake a detailed histotype-specific analysis to evaluate the actual versus expected effects of individual LOY components to assess whether the mechanism of action of these components is solely by ovulation suppression.

To investigate the effects of LOY and its components on EOC, we pooled data from 25 case-control studies from the Ovarian Cancer Association Consortium (OCAC). Our goals were to (1) quantify the LOY-EOC association overall and for individual histotypes, (2) assess the impact of LOY definition on the LOY-EOC relationship, and (3) determine whether the relationship between LOY components and EOC is beyond ovulation suppression.

## Methods

### Study population

This study included 25 case-control studies (Table 1)^16–42^ from OCAC.^43^ Participants provided informed consent for original studies, whose protocols were approved by their respective Institutional Review Boards.

### Study variables and LOY calculation

OCAC’s harmonized core data provided LOY component variables: age at last menstrual period (LMP) before diagnosis (cases) or interview (controls), age at menarche, number of pregnancies, number of full-term births, and total durations of pregnancy, breastfeeding, and OC use.

**Figure 1:**
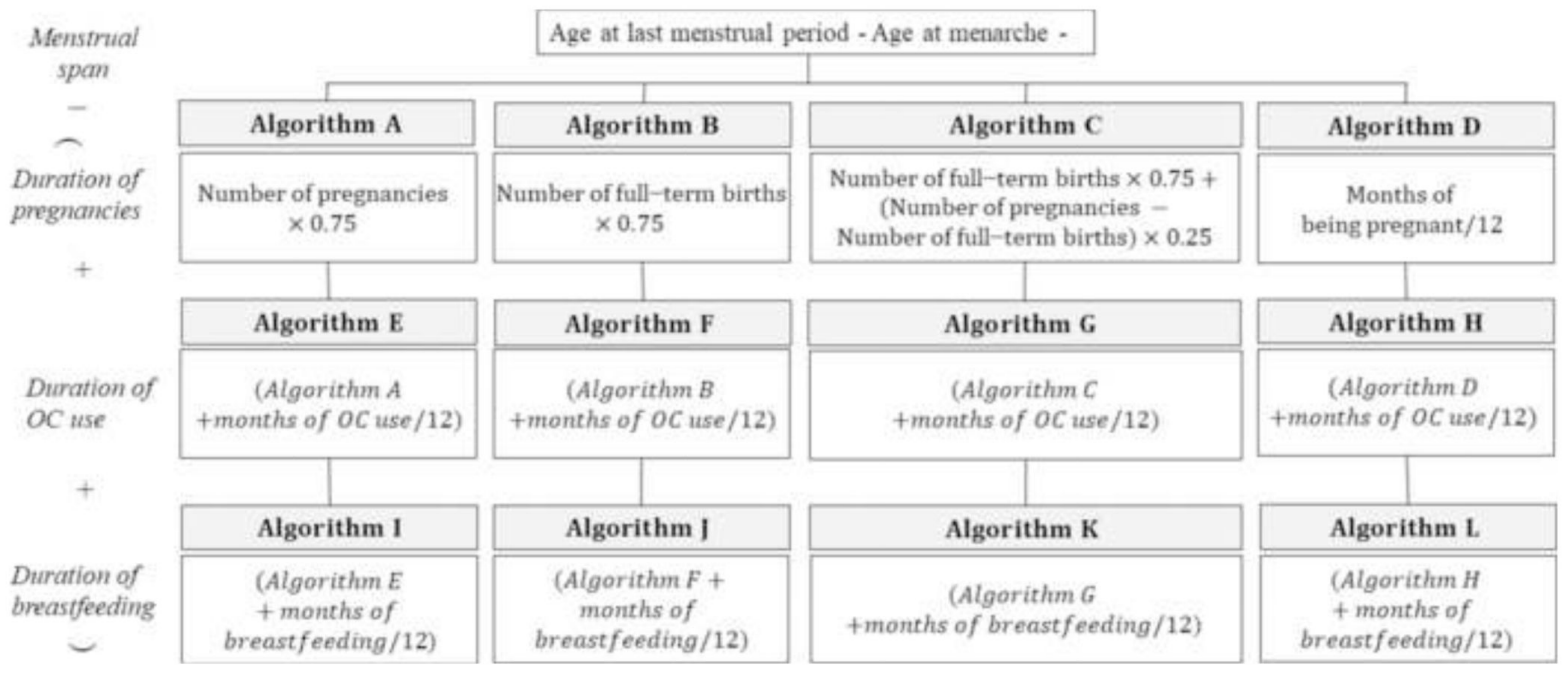
Flow chart for algorithms to calculate lifetime ovulatory years OC, oral contraceptive.

LOY was calculated with 12 algorithms (Supplementary Table 1)^8^ using the formula:

LOY=menstrualspan−yearsofanovulation

where “menstrual span” was calculated from age at LMP minus age at menarche. The algorithms were divided into four classes based on how “years of anovulation” was defined (Figure 1).

**Figure 2:**
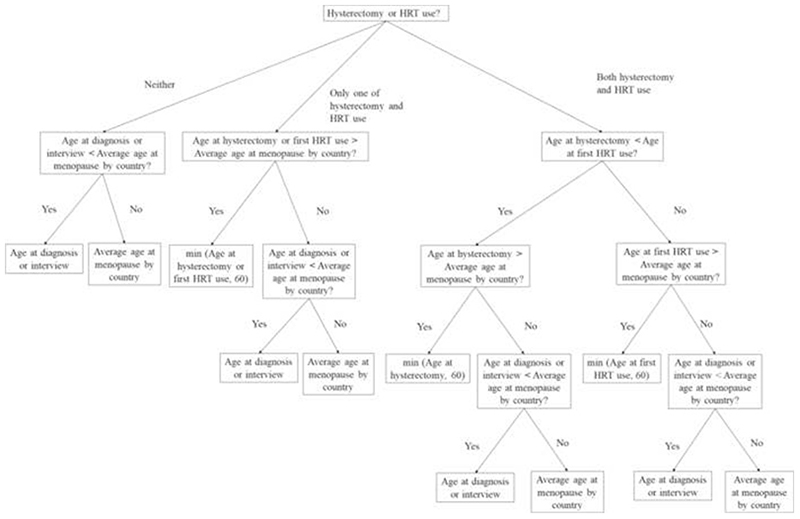
Flow chart for imputation of age at last menstrual period (LMP). HRT, hormone replacement therapy.

Seven studies recorded age at LMP (cases: 6881 (32.4% of total), controls: 8316 (31.7% of total)). For the remaining studies, we imputed age at LMP (Figure 2)^44^ and assessed the imputation algorithm by comparing actual versus imputed age at LMP for the seven sites (Supplementary Table 2). Sites with 50+% missing values in any LOY component except age at LMP were excluded from algorithms using those components (Supplementary Table 3).^45,46^

Variables considered *a priori* as potential confounders included age at diagnosis (cases) or interview (controls), race, education, body mass index (BMI) 1-year to 5-years prior, family history of ovarian or breast cancer in a first-degree relative, smoking status, history of endometriosis, and tubal ligation.

### Statistical analyses

#### Assessment of study heterogeneity

We used random effects meta-analysis to assess inter-study LOY-EOC heterogeneity. Because we observed no substantive heterogeneity (Supplementary Figure 1), we used the pooled data set adjusted for study site for all analyses.

#### Correlations between LOY values among algorithms and between LOY and LOY components

We used Pearson’s correlation to assess pairwise correlations of LOY calculated among algorithms limiting analyses to observations with complete data for each algorithm in the pairwise comparison. Pearson’s correlation was also used to assess the correlations of individual components with LOY calculated by each algorithm.

#### Estimation of LOY-EOC association

Multivariable logistic regression was used to estimate odds ratios (ORs) with 95% confidence intervals (95% CIs) for the association between LOY and EOC overall and by histotype. Models were adjusted for study site, age at diagnosis or interview, race, education, BMI, smoking status, and family history; inclusion of tubal ligation and endometriosis in models did not alter findings and were omitted from final models. Because OCAC only recorded total months of breastfeeding across all live births and not months per breastfeeding episode, to account for return of ovulation once food is introduced typically at 6 months, we performed sensitivity analyses replacing breastfeeding duration with either (1) number of live births times the average duration of breastfeeding per live birth if the average duration was less than 6 months, or (2) number of live births times 6 months if the average duration was 6 months or greater. Similar sensitivity analyses were performed for algorithms containing a term for breastfeeding duration (Algorithms I-L). Sensitivity analyses were performed with multiple imputation by chained equation (MICE) to assess the effect of missing values on LOY-EOC associations^47^ including the same covariates as main models. Nested imputations were done for number of pregnancies, number of full-term births, duration of breastfeeding, and duration of OC use using the binary variables of ever pregnant, ever breastfed, and OC use, respectively. Imputations were done five times with auxiliary variables defined as Pearson’s correlation larger than 0.4.^48^ Sensitivity analyses also examined limiting models to population-based studies and using only observations with complete data for all variables.

To assess the relationship between LOY and EOC histotypes, we present results using algorithm K because this algorithm most closely reflects lifetime ovulatory years accounting for OC use, pregnancy type, and breastfeeding.

Prior studies suggest that the relationship between LOY and EOC may not be linear;^49^ thus, we constructed models using LOY and log(LOY). Because log(LOY) did not improve model fit when included with LOY and models with LOY alone provided a better fit than those with log(LOY) alone, we report only analyses using LOY.

#### Estimation of EOC risk related to LOY components: observed versus expected estimates

The association of each LOY component and EOC risk overall and separately for each histotype was estimated using multivariable logistic regression adjusted for study site, age at diagnosis (cases) or interview (controls), race, education, BMI one to five years prior to diagnosis/interview, smoking status, family history, and other LOY components.

To assess whether each component acts through ovulation suppression alone, we compared expected beta coefficient to actual estimates obtained from regression models.^7^ Based on the “incessant ovulation” hypothesis, one year of ovulation suppression should have the same effect on the log odds of EOC regardless of origin. Thus, if we assign one as the expected beta coefficient for age at LMP per year (indicating that a one-year increase in LMP, which would increase LOY by 1, would increase the log odds by 1), then the expected beta coefficient for age at menarche per year would be -1 because each additional year increase would decrease LOY by one year and hence decrease the log odds by 1. Similarly, the expected beta coefficients for OC use per year, number of incomplete pregnancies (assumed to be 3 months or 0.25 years), number of full-term births (assumed to be 9 months or 0.75 years), and breastfeeding per year would be -1, -0.25, -0.75 and -1, respectively.

We then computed the relative coefficients, defined as the actual coefficients from regression models divided by the actual coefficient of age at LMP. This set the relative coefficient for age at LMP to 1, just as in the expected model. This enabled us to compare the actual relative coefficient estimates coefficients to their expected counterparts. To assess the significance of individual components, χ2 statistics and p-values were obtained from the likelihood-ratio test for the removal of each component from the full model. Sensitivity analyses examined limiting models to population-based studies and using only observations with complete data for all variables.

All statistical tests were two-sided and performed in Stata/SE version 16.1 (StataCorp, College Station, TX).

## Results

### Study population

Among the 25 studies, there were 26,204 controls and 21,267 cases (Table 2). Compared to controls, cases were more likely to have a family history of breast or ovarian cancer, a history of endometriosis, be hysterectomized, and be obese/overweight. Controls were more likely to have never smoked, be pre-menopausal, and have had a tubal ligation. Cases reported a shorter total duration of OC use and breastfeeding, and fewer total pregnancies.

### LOY estimations and correlations

**Figure 3:**
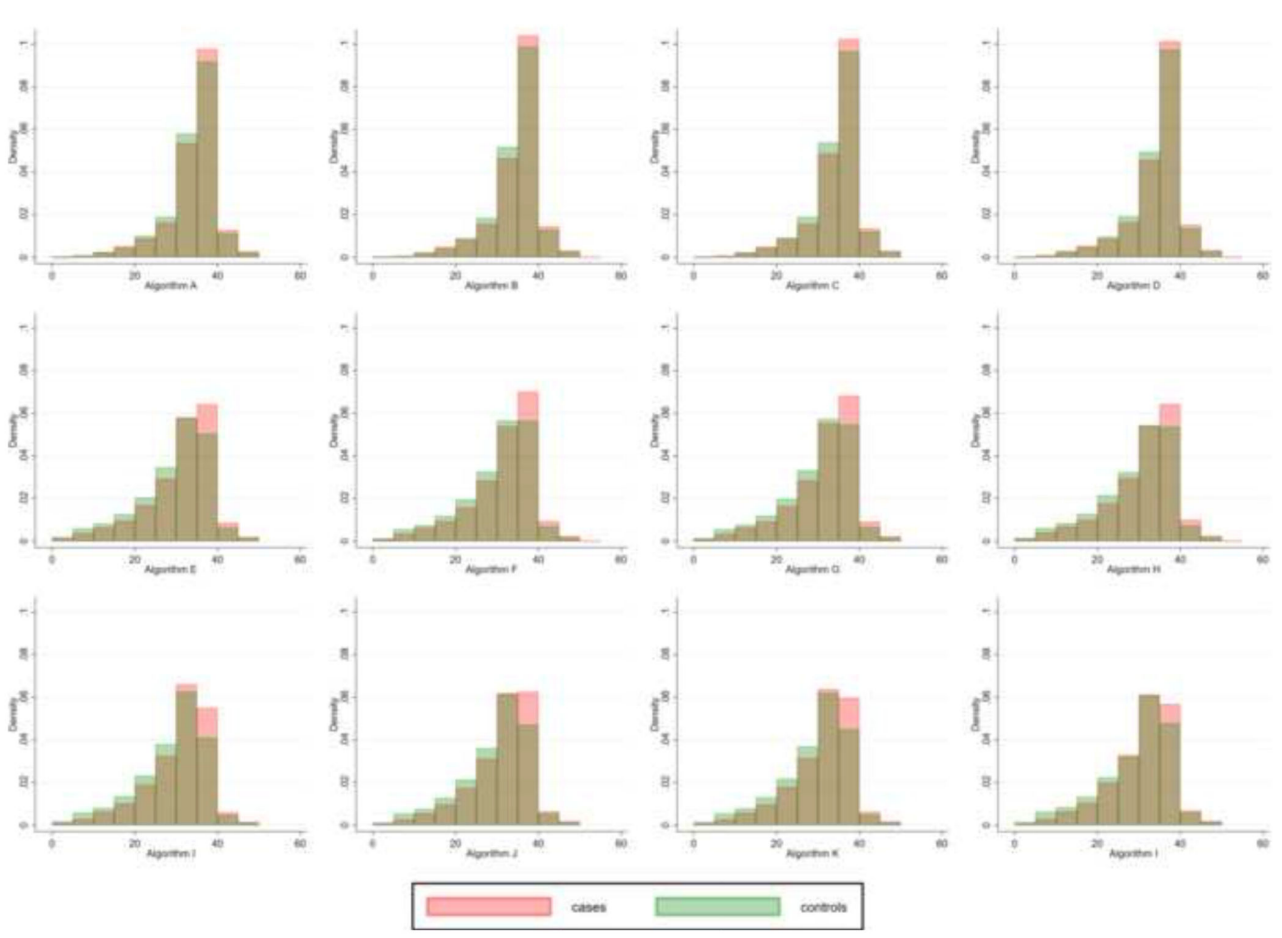
Distribution of lifetime ovulatory years calculated from 12 different algorithms

Among the 12 algorithms, median LOY ranged from 31.67 [interquartile range (IQR) 25.50-35.20] to 35.75 [IQR 32.50-37.50] years (Figure 3 and Supplementary Table 4). Pairwise LOY correlations ranged from 0.75 between the algorithms in the first class (inclusive of pregnancies only) and the third class (inclusive of pregnancies, OC use, and breastfeeding,) to ≥0.99 for correlations within the same class (Supplementary Table 5). Correlations between individual components and LOY are presented in Supplementary Table 6. As algorithm complexity increased, correlations between age at LMP and LOY decreased. OC duration was moderately negatively correlated with LOY (rho range: -0.68 to -0.69); correlations between the other components and LOY were low.

### Estimation of LOY-EOC association (Table 3)

ORs for LOY per year increase across the 12 algorithms ranged from 1.014 (95%CI 1.009-1.020) to 1.044 (95%CI 1.041-1.048). Associations with LOY calculated from the third class of algorithms (inclusive of pregnancies, OC use, and breastfeeding) were not changed when months of breastfeeding were truncated at six for participants reporting more than six months per birth (data not shown). LOY associations remain unchanged when adjusting models in the first class of algorithms (which included only pregnancies) for OC and breastfeeding duration, as well as when adjusting the second class of algorithms (which included pregnancies and OC duration) for breastfeeding duration (data not shown). Sensitivity analyses with multiple imputations of missing values did not alter LOY-EOC associations (Table 3). Sensitivity analyses limited to populationbased studies and those limited to observations with complete data also did not alter the LOY-EOC association (data not shown).

### Estimation of EOC risk related to LOY components: observed versus expected estimates (Table 4)

Individual components in LOY, except for age at menarche, were associated with EOC. There were substantial deviations between relative estimated coefficients and expected estimates for each component. The estimated coefficient of OC use per year was 4.45 times larger than expected, while estimates for pregnancies were 11-15-fold greater than expected regardless of pregnancy type. Estimated coefficient of breastfeeding per year was -13.45, instead of the expected -1. Results were similar when truncating breastfeeding at 6 months per full-term birth, when limiting analyses to population-based studies, and when limiting analyses to observations with complete data (data not shown).

### Histotype-specific estimation for LOY and individual components: observed versus expected estimates (Table 5)

LOY was associated with invasive high-grade serous (HGSOC; OR per year 1.054, 95%CI 1.048-1.061), low-grade serous (LGSOC; OR 1.040, 95%CI 1.019-1.061), endometrioid (OR 1.065, 95%CI 1.053-1.076), and clear cell (OR 1.098, 95%CI 1.079-1.117), but not mucinous EOC (OR 1.006, 95% CI 0.992-1.019). Except for breastfeeding, estimated coefficients of LOY components were close to expected for HGSOC. In contrast, estimated coefficients of individual components, except for age at menarche, were larger than the expected for LGSOC, endometrioid, and clear cell cancers.

## Discussion

Pooling data from 25 case-control studies, we show a positive association between LOY and EOC, with each year of ovulation associated with a 4% increase in risk. We also found a positive association between LOY and HGSOC, LGSOC, endometrioid, and clear cell EOC but not with mucinous tumors. These LOY-EOC associations were not altered when using different algorithms to compute LOY or when imputing missing data. We further found that LOY components, except age at menarche, were associated with EOC, with the magnitude of these associations varying substantially from expectation if their mechanism of action were solely ovulation suppression. There was also notable heterogeneity in these component-specific findings among EOC histotypes. Together, these data suggest that reproductive factors comprising LOY exert their effects through means beyond ovulation suppression and those relationships vary by EOC subtype.

Most prior studies report a positive relationship between LOY and EOC.^2,7–14,50–63^ Differences in LOY definitions among studies make it challenging to compare specific findings across studies. In the present study, we defined LOY from available harmonized data using 12 algorithms. Like the Polish Cancer study^8^ (one of the 25 studies in this analysis), we found a high correlation for LOY among algorithms, although point estimates varied depending on the algorithm. When assessing overall EOC per 1-year increase in LOY, estimates ranged from 1.01-1.04, which is similar to estimates reported by the US Nurses’ Health Study (1976-2006) (NHS) and Nurses’ Health Study II (1989-2005) (NHS II) (OR=1.07; 95% CI 1.05 -1.08).^10^ While it is reassuring that our results are similar to previous work, because each study used different LOY algorithms and units of presentation (e.g., quartiles, ovulatory cycles, etc.)^15^, a direct comparison of estimated magnitudes is not possible. A standardized definition of LOY would facilitate cross-study comparisons and allow for more robust inter-study analyses. Our findings confirm that among algorithms that account for menstrual span, number of pregnancies, total duration of OC use, and total duration of breastfeeding, point estimates for the LOY-EOC relationship are similar. Defining LOY using these factors would facilitate inter-study analyses.

We report differences in the association of LOY with EOC subtypes. We report a positive association between LOY and both HGSOC and LGSOC. While previous studies have reported a positive association between LOY and risk of serous tumors,^2,10–15^ only OC3^14^ reported results separately for HGSOC, also finding a positive association. Separating serous EOC analyses is important because HGSOC and LGSOC are distinct diseases.^64,65^ Also consistent with most^10–14^ but not all previous studies^2,15^ we found positive associations between LOY and clear cell and endometrioid but not mucinous tumors. These results are consistent with epidemiologic evidence that suggests a different risk-factor profile for mucinous EOC.^3,66^

Results regarding the associations between LOY components and EOC appeared consistent with previous studies.^7,8,10,12,58,62^ Beyond considering statistical significance, our study also compared the magnitudes of each component’s effect on EOC risk and found the actual magnitudes varied substantially from expectation.^7^ Based on the “incessant ovulation” hypothesis,^6^ women with the same LOY should have the same estimated risk if ovulation is the only etiologic mechanism underlying the relationship between the components of LOY and EOC. However, consistent with two case-control studies,^7,62^ we show that pregnancy, OC use, and breastfeeding are associated with stronger protective effects than would be expected based on ovulation suppression alone. Moreover, the protection from one year of pregnancy, whether complete or incomplete, was substantially greater than that of one year of OC use.^7^ Together, these data imply that mechanisms beyond ovulation suppression, such as hormonal alterations^67,68^ or inflammation,^69^ contribute to the LOY-EOC association. They further imply differences in the mechanisms whereby individual LOY components impact EOC risk, especially for non-HGSOC subtypes, suggesting that a model of EOC risk incorporating just LOY and not its component parts would be insufficient in fully capturing the effects of exposure to LOY components.

Our results indicate heterogeneity in the associations between LOY components and histotype-specific risk. Notably, except for breastfeeding, the estimated coefficients for HGSOC were close to expected if only ovulation suppression underlies the component-HGSOC relationship. This suggests that ovulation may be the primary etiologic mechanism for HGSOC; however, because HGSOC is believed to arise in the fimbriated end of the fallopian tube and not the ovary^70–72^ ovulation effects must extend beyond ovarian surface epithelium trauma, as originally proposed by Fathalla.^6^ Notably, during ovulation, fallopian tube fimbria come in close proximity to the site of ovulation, directly exposing the fimbria to ovarian follicular fluid. *In vitro* studies show that normal fallopian tube epithelia exposed to follicular fluid aspirates develop TP53 mutations, a hallmark of HGSOC.^73^ Moreover, follicular fluid has both mutagenic and tumorigenic effects facilitating the full transformation process for developing HGSOC from the fallopian tube.^74–77^ Thus, follicular fluid may be the link between greater number of ovulations and HGSOC.

In contrast to HGSOC, factors beyond ovulation suppression underlie the link between LOY and other histotypes. For LGSOC, endometrioid and clear cell histotypes, we found that actual coefficient estimates were substantially larger than expected for OC use, pregnancies, and breastfeeding. This suggests that other mechanisms, such as increased progestin exposure,^78^ may play a role in the protective effects of these factors.

While we did not find any association between LOY and mucinous EOC, we report associations for several LOY components. Thus, factors other than ovulation may be driving mucinous carcinogenesis. Moreover, the relationship between LOY components and mucinous disease varied from that of other histotypes. Together, these observations suggest that factors underlying the relationship between exposures and EOC vary based on histotype and confirm the unique origin of mucinous cancers.^79,80^

The major strength of our work was pooling 25 case-control studies, allowing us to estimate more precisely the LOY-EOC association overall and by histotype. The large data set also enabled comparison of different LOY definitions and their impact on the LOY-EOC relationship. For LOY components, the sample size enabled us to separate the effects of ovulation suppression from other potential etiologic mechanisms. The range of studies from four continents and nine countries supports the generalizability of our findings.

Despite these strengths, there are several limitations. Because all but two studies^25,42^ employed a retrospective case-control design, recall and selection bias are always a concern. Regardless of study design limitations, our estimates were consistent with previous prospective studies, including the NHS and NHS II study^10^ and the OC3 pooled analysis of prospective studies.^14^ We made some assumptions about LOY components that may impact results. If age at LMP was unknown, we imputed it using an algorithm based on average age at menopause by country, age at first HRT use, or age at hysterectomy. We compared the observed and imputed age at LMP from seven sites, conducted sensitivity analyses using LOY calculated from the imputed value for those sites, and noted no differences in observed associations. To prevent overestimating the duration of anovulation from breastfeeding, we repeated analyses capping women at six months of breastfeeding per live birth. Results were unchanged.

In conclusion, increasing LOY is associated with increased EOC risk, as well as the risk of HGSOC, LGSOC, endometrioid, and clear cell histotypes. Although point estimates varied slightly, the association between LOY and EOC was not altered when LOY was calculated in different ways using core components. Our study also indicated heterogeneity in the expected estimated coefficients of each LOY component on histotype-specific EOC. Together, our findings suggest that ovulation suppression is not the sole mechanism whereby reproductive factors affect EOC overall and for non-HGSOC histotypes. Identifying these mechanisms and understanding their individual and joint roles can provide deeper insight into disease etiology and potential riskreducing approaches.

## Supplementary Material

Supplementary Material

## Figures and Tables

**Table 1:** Characteristics of the 25 case-control Studies from the Ovarian Cancer Association Consortium, conducted in Asia, Australia, Europe, and North America from 1989 to present and included in the lifetime ovulatory years (LOY) analyses

Study	Region	Study Name	Study Period	Cases Type	Method of Data Collection	Age (years), mean (SD)	Controls, n (%)	Cases, n(%)
AUS^16^	Australia	Australian Ovarian Cancer Study/Australian Cancer Study	2002-2006	Population-based	Self-completed questionnaire	56.88 (12.28)	1506 (43.2)	1984 (56.8)
BAV^17^	Germany	Bavarian Ovarian Cancer Cases and Controls	2002-2006	Hospital/Clinic-based	Interview	57.31 (13.77)	629 (47.9)	684 (52.1)
CON^18^	USA	Connecticut Ovarian Cancer Study	1998-2003	Population-based	Interview	55.27 (11.04)	551 (52.6)	497 (47.4)
DOV^19^	USA	Diseases of the Ovary and their Evaluation	2002-2009	Population-based	Interview	55.78 (9.26)	1849 (54.2)	1562 (45.8)
GER^20^	Germany	German Ovarian Cancer Study	1993-1996	Population-based	Self-completed questionnaire	55.07 (12.24)	533 (67.4)	258 (32.6)
HAW^21^	USA	Hawaii Ovarian Cancer Case-Control Study	1993-2008	Population-based	Interview	54.98 (14.28)	1103 (55.2)	895 (44.8)
HOP^22^	USA	Hormones and Ovarian cancer PrEdiction	2003-2009	Population-based	Interview	58.66 (12.52)	1802 (68.3)	836 (31.7)
JPN^23^	Japan	Hospital-based Research Program at Aichi Cancer Center	2001-2005	Hospital/Clinic-based	Interview	52.36 (11.17)	233 (60.5)	152 (39.5)
MAY^24^	USA	Mayo Clinic Ovarian Cancer Case-Control Study	1999-2018	Hospital/Clinic-based	Interview	60.51 (13.58)	2299 (55.5)	1846 (44.5)
*MCC^25^	Australia	Melbourne Collaborative Cohort Study	1990-2008	Defined Cohort	Self-completed questionnaire	64.07 (9.62)	471 (73.1)	173 (26.9)
NCO^26^	USA	North Carolina Ovarian Cancer Study	1999-2008	Population-based	Interview	55.28 (11.53)	1085 (47.6)	1195 (42.4)
NEC^27^	USA	New England Case Control Study	1992-2003	Population-based	Interview	53.54 (12.35)	2100 (50.0)	2075 (49.7)
NJO^28^	USA	New Jersey Ovarian Cancer Study	2002-2008	Population-based	Interview	61.48 (11.60)	458 (65.9)	237 (34.1)
NTH^29,30^	Netherlands	Nijmegen Ovarian Cancer Study	1997-2008	Population-based	Self-completed questionnaire	55.90 (10.79)	600 (69.4)	265 (30.6)
OVA	Canada	Ovarian Cancer in Alberta and British Columbia	2002-2012	Population-based	Self-completed questionnaire 2002-2004; interview 2004-2012	56.81 (10.62)	2698 (62.2)	1637 (37.8)
POL^31^	Poland	Polish Ovarian Cancer Case Control Study	2000-2003	Population-based	Interview	55.70 (10.62)	1128 (79.3)	294 (20.7)
SON^32^	Canada	Southern Ontario Ovarian Cancer Study	1989-1993	Population-based	Interview	56.86 (11.97)	564 (55.6)	450 (44.4)
STA^33^	USA	Family Registry for Ovarian Cancer AND Genetic Epidemiology of Ovarian Cancer	1997-2001	Population-based	Interview	47.77 (10.07)	567 (46.0)	665 (54.0)
SWH^34^	China	Shanghai Women’s Health Study	1996- present	Defined Cohort	Interview	53.36 (9.70)	986 (86.6)	152 (13.4)
TBO^35^	USA	Tampa Bay Ovarian Cancer Study	2000- present	Population-based	Interview	60.53 (10.85)	205 (41.8)	285 (58.2)
TOR^36^	Canada	Familial Ovarian Tumour Study (FOTS) AND Health Watch (HW)	1995-1999 and 2000-2003	Population-based	Interview	56.62 (12.77)	322 (21.6)	1167 (78.4)
UCI^37^	USA	University California Irvine Ovarian Study	1993-2005	Population-based	Interview	54.29 (13.17)	614 (49.1)	636 (50.9)
UKO^38^	UK	United Kingdom Ovarian cancer Population Study	2006-2010	Hospital/Clinic-based	Interview	63.06 (8.93)	1182 (58.5)	839 (41.5)
USC^39–41^	USA	Los Angeles County Case-Control Studies of Ovarian Cancer	1992-2009	Population-based	Interview	55.07 (12.41)	2595 (52.2)	2380 (47.8)
^a^VTL^42^	USA	VITamins And Lifestyle Cohort Study	2000-2010	Defined Cohort	Self-completed questionnaire	68.19 (7.62)	124 (54.6)	103 (45.4)
Total						56.55 (12.20)	26204 (55.2)	21267 (44.8)

a Employed a nested-case control study design within a cohort study

**Table 2:** Characteristics of ovarian cancer cases and controls included in the lifetime ovulatory years (LOY) analyses

Variables	Control, n (%) N= 26204	Case, n (%) N=21267
Age, years, mean (SD)	56.51 (12.06)	56.59 (12.36)
Race		
White	22,586 (86.2)	18,685 (87.9)
Black	566 (2.2)	460 (2.2)
Asian	2,019 (7.7)	1,227 (5.8)
Other	775 (3.0)	692 (3.3)
Unknown	258 (1.0)	203 (1.0)
Education		
Less than high school	2,857 (10.9)	2,512 (11.8)
Completed high school	6,508 (24.8)	5,309 (25.0)
Completed some college	5,573 (21.3)	4,849 (22.8)
Completed college or university bachelor’s degree	4,727 (18.0)	3,344 (15.7)
Completed graduate or professorial degree	3,139 (12.0)	2,271 (10.7)
Unknown	3,400 (13.0)	2,982 (14.0)
Body Mass Index (BMI) at 18, kg/m^2^		
<18.5	2,637 (10.1)	2,008 (9.4)
18.5-24.9	10,697 (40.8)	8,809 (41.4)
25-29.9	992 (3.8)	1,002 (4.7)
≥30	310 (1.2)	353 (1.7)
Unknown	11,568 (44.2)	9,095 (42.8)
Body Mass Index 1 or 5 years prior, kg/m^2^		
<18.5	286 (1.1)	274 (1.3)
18.5-24.9	7,472 (28.5)	5,672 (26.7)
25-29.9	4,541 (17.3)	3,570 (16.8)
≥30	3,074 (11.7)	3,021 (14.2)
Unknown	10,831 (41.3)	8,730 (41.1)
Smoking Status		
Never Smoker	13,311 (50.8)	10,106 (47.5)
Former Smoker	2,900 (11.1)	2,682 (12.6)
Current Smoker	7,449 (28.4)	5,930 (27.9)
Unknown	2,544 (9.7)	2,549 (12.0)
Family History of Breast or Ovarian Cancer in first-relative		
No	16,038 (61.2)	11,574 (54.4)
Yes	1,569 (6.0)	1,808 (8.5)
Unknown	8,597 (32.8)	7,885 (37.1)
Tubal ligation	
No	16,351 (62.4)	15,035 (70.7)
Yes	5,138 (19.6)	3,345 (15.7)
Unknown	4,715 (18.0)	2,887 (13.6)
Menopausal status		
Pre/peri-menopausal	8,206 (31.3)	5,775 (27.2)
Post-menopausal	16,749 (63.9)	14,422 (67.8)
Unknown	1,249 (4.8)	1,070 (5.0)
Endometriosis		
No	18,294 (69.8)	15,128 (71.1)
Yes	1,291 (4.9)	1,615 (7.6)
Unknown	6,619 (25.3)	4,524 (21.3)
Hysterectomy pre-diagnosis (cases) or interview (controls)		
No	20,969 (80.0)	14,562 (68.5)
Yes	4,004 (15.3)	5,008 (23.6)
Unknown	1,231 (4.7)	1,697 (8.0)
Hormone replacement therapy		
No	15,547 (59.3)	13,097 (61.6)
Yes	7,472 (28.5)	5,921 (27.8)
Unknown	3,185 (12.2)	2,249 (10.6)
Components of lifetime ovulatory years		
Age at last menstrual period before diagnosis or interview, n(%)	26,204 (100.0)	21,267 (100.0)
mean (SD)	48.77 (6.03)	48.84 (6.4)
Age at Menarche, n(%)	25,255 (96.4)	20,101 (94.5)
mean (SD)	12.91 (1.7)	12.79 (1.6)
Duration of Oral Contraceptive Use, months, n(%)	24,948 (95.2)	19,762 (92.9)
mean (SD)	52.12 (71.3)	37.42 (59.3)
Number of Pregnancies, regardless of outcome, n(%)	25,429 (97.0)	20,429 (96.1)
mean (SD)	2.75 (1.8)	2.40 (1.9)
Total number of months of being pregnant, regardless of outcome(s), n(%)	14,438 (55.1)	12,195 (57.3)
mean (SD)	21.42 (22.3)	16.39 (17.6)
Total number of full-term births, n(%)	22,835 (87.1)	18,304 (86.1)
mean (SD)	2.13 (1.5)	1.85 (1.6)
Total months of breastfeeding, n(%)	18,578 (70.1)	13,619 (64.0)
mean (SD)	9.52 (14.4)	6.86 (13.1)
Behavior and Histotypes		
Invasive	-	17,465 (82.1)
High-Grade-Serous	-	7,492 (71.8)
Low-Grade Serous	-	513 (4.9)
Serous (Unknown Grade)	-	2,418 (23.2)
Endometrioid	-	2,536(14.5)
Mucinous	-	1,134 (6.5)
Clear cell	-	1,310 (7.5)
Mixed	-	566 (3.2)
Others	-	1,496 (8.6)
Low Malignant Potential (Borderline Tumors)	-	3,602 (16.9)
Unknown behavior	-	200 (0.9)

**Table 3:** Odds ratio for ovarian cancer per lifetime ovulatory year using complete data and full data with imputation

	Main analyses^b^ (complete data only)			Sensitivity analyses^b^ (includes imputed data)
	Controls	Cases	Odds Ratio^a^ (95% Confidence Interval)	Odds Ratio^a^ (95% Confidence Interval)
The first class of algorithms – anovulation due to pregnancy				
Algorithm A	25,081	20,046	1.018 (1.013, 1.022)	1.015 (1.011, 1.020)
Algorithm B	22,519	18,013	1.014 (1.009, 1.020)	1.012 (1.007, 1.017)
Algorithm C	22,509	18,003	1.016 (1.011, 1.021)	1.014 (1.009, 1.019)
Algorithm D^c^	13,617	10,689	1.016 (1.010, 1.023)	1.009 (1.003, 1.016)
The second class of algorithms – anovulation due to pregnancy and OC use				
Algorithm E	24,480	19,323	1.044 (1.041, 1.048)	1.043 (1.039, 1.046)
Algorithm F^d^	22,316	17,772	1.043 (1.039, 1.046)	1.042 (1.039, 1.046)
Algorithm G^d^	22,306	17,762	1.043 (1.040, 1.047)	1.043 (1.039, 1.047)
Algorithm H^c,d^	13,515	10,576	1.043 (1.039, 1.048)	1.041 (1.036, 1.045)
The third class of algorithms – anovulation due to pregnancy, OC use, and breastfeeding				
Algorithm I^e^	14,900	11,829	1.041 (1.036, 1.045)	1.047 (1.043, 1.051)
Algorithm J^f^	14,902	11,339	1.041 (1.036, 1.045)	1.046 (1.042, 1.050)
Algorithm K^f^	14,900	11,329	1.041 (1.036, 1.046)	1.046 (1.042, 1.050)
Algorithm L	8,473	6,498	1.040 (1.034, 1.046)	1.047 (1.042, 1.052)

a Adjusted for study site, age, race (White, Black, Asian, other, unknown), education (less than high school, completed high school, completed some college, completed college or university bachelor’s degree, completed graduate or professorial degree, unknown), body mass index 1 or 5 years prior (underweight, normal, overweight, obese, unknown), smoking status (never, former, current, unknown), and family history (yes, no, unknown).

b Main analyses included participants without missing values in any component for LOY calculation; sensitivity analyses included all participants with imputation.

c TBO was excluded from the sensitivity analyses due to limited numbers within site to impute missing values.

d MCC was excluded from the sensitivity analyses due to limited numbers within site to impute missing values.

e NTH was excluded from the sensitivity analyses due to fail to converge on observed data.

f NTH was excluded from the sensitivity analyses due to limited numbers within site to impute missing values.

**Table 4:** Odds ratios, expected beta coefficients, and normalized beta coefficients for ovarian cancer by individual components of lifetime ovulatory years

	Odds Ratio^a^	95% Confidence Interval	Expected estimate of coefficient	Normalized coefficient^b^	P-value for removal of component from model
Age at last menstrual period before diagnosis or interview					
per year	1.011	1.004, 1.019	1 (defined)	1	0.004
Age at Menarche					
per year	1.002	0.985, 1.018	-1	0.13	0.86
Duration of Oral Contraceptive Use, years					
per year	0.950	0.945, 0.956	-1	-4.45	<0.001
Number of incomplete pregnancies					
per pregnancy	0.968	0.944, 0.992	-0.25	-2.89	0.009
Number of full-term births					
per pregnancy	0.877	0.857, 0.897	-0.75	-11.53	<0.001
Total years of breastfeeding					
per year	0.858	0.816, 0.901	-1.0	-13.45	<0.001

a adjusted for study site, age, race (white, black, Asian, other and unknown), education (less than high school, completed high school, completed some college, completed college or university bachelor’s degree, completed graduate or professorial degree, unknown), body mass index 1 or 5 years prior (underweight, normal, overweight, obese, unknown), smoking status (never, former, current, unknown), family history (yes, no, unknown), and other components of lifetime ovulatory cycles in the model.

b Normalized to the beta coefficient of age at last menstrual period.

**Table 5:** Odds ratios, expected beta coefficients, and normalized beta coefficients for ovarian cancer histotypes by individual components of lifetime ovulatory years

Expected estimate of coefficient		Low malignant potential N=2014		Invasive high-grade serous N=4139		Invasive low-grade serous N=282		Invasive endometrioid N=1322		Invasive mucinous N=602		Invasive clear cell N=547	
		OR^a^ (95% CI)	β^c^	OR^a^ (95% CI)	β^c^	OR^a^ (95% CI)	β^c^	OR^a^ (95% CI)	β^c^	OR^a^ (95% CI)	β^c^	OR^a^ (95% CI)	β^c^
Lifetime ovulatory years^b,d^													
per year		1.015 (1.007, 1.024)	-	1.054 (1.048, 1.061)	-	1.040 (1.019, 1.061)	-	1.065 (1.053, 1.076)	-	1.006 (0.992, 1.019)	-	1.098 (1.079, 1.117)	-
Age at last menstrual period before diagnosis or interview													
per year	1	0.981 (0.967, 0.995)	1	1.056 (1.044, 1.069)	1	1.010 (0.976, 1.044)	1	1.031 (1.013, 1.049)	1	0.977 (0.955, 1.000)	1	1.086 (1.057, 1.117)	1
Age at Menarche													
per year	-1	1.027 (0.995, 1.059)	-1.360	1.000 (0.977, 1.023)	-0.008	0.961 (0.891, 1.037	-4.157	1.003 (0.967, 1.041)	0.093	1.076 (1.023, 1.132)	-3.208	0.948 (0.867, 1.002)	-0.643
Duration of Oral Contraceptive Use, years													
per year	-1	0.973 (0.962, 0.983)	1.441	0.948 (0.940, 0.956)	-0.975	0.953 (0.929, 0.978)	-5.042	0.928 (0.914, 0.942)	-2.472	0.973 (0.955, 0.991)	1.186	0.925 (0.904, 0.947)	-0.942
Number of incomplete pregnancies													
per pregnancy	-0.25	0.998 (0.955, 1.044)	0.082	0.987 (0.953, 1.021)	-0.245	0.889 (0.782, 1.010)	-12.484	0.926 (0.872, 0.983)	-2.533	0.944 (0.867, 1.027)	2.516	0.853 (0.774, 0.940)	-1.924
Total number of full-term births													
per pregnancy	-0.75	0.824 (0.785, 0.864)	10.085	0.938 (0.909, 0.967)	-1.170	0.915 (0.821, 1.021)	-9.356	0.740 (0.699, 0.784)	-9.884	0.883 (0.819, 0.953)	5.417	0.630 (0.573, 0.692)	-5.583
Total years of breastfeeding													
per year	-1	0.963 (0.867, 1.069)	1.970	0.827 (0.771, 0.886)	-3.467	0.808 (0.627, 1.041)	-22.533	0.830 (0.727, 0.948)	-6.120	1.010 (0.858, 1.190)	-0.455	0.893 (0.723, 1.103)	-1.370

CI, confidence interval; OR odds ratio; β, estimated coefficient.

a adjusted for study site, age, race (white, black, Asian, other and unknown), education (less than high school, completed high school, completed some college, completed college or university bachelor degree, completed graduate or professorial degree, unknown), body mass index 1 or 5 years prior (underweight, normal, overweight, obese, unknown), smoking status (never, former, current, unknown), family history (yes, no, unknown), and other components of lifetime ovulatory cycles in the model.

b adjusted for study site, age, race (white, black, Asian, other and unknown), education (less than high school, completed high school, completed some college, completed college or university bachelor’s degree, completed graduate or professorial degree, unknown), body mass index 1 or 5 years prior (underweight, normal, overweight, obese, unknown), smoking status (never, former, current, unknown), and family history (yes, no, unknown).

c normalized to the beta coefficient of age at last menstrual period

d Using algorithm K with complete data: (age at last menstrual period – age at menarche) – years of OC use – (0.25*number of incomplete pregnancies) – (0.75*number of full-term births) – years of breastfeeding. This algorithm was chosen because it most closely accounts for expected ovulation suppression due to pregnancies, OC use, and breastfeeding.

## Data Availability

The data generated in this study are not publicly available due to restrictions of some included studies’ informed consent. The corresponding author will facilitate access to data through existing data request processes for OCAC.
