# Supplementary material for: Lifetime ovulatory years and risk of epithelial ovarian cancer: a multinational pooled analysis
Source: J Natl Cancer Inst. Author manuscript; Available in PMC 2023 Oct 27. (PMC10165492; doi:10.1093/jnci/djad011)
Supplement: Supplementary Material [file EMS189178-supplement-Supplementary_Material.pdf]

Supplementary Material

Supplementary Table 1. Algorithms to calculate lifetime ovulatory years (LOY). Each algorithm starts with menstrual span defined as age at last menstrual period minus age a menarche. The first class of algorithms exclude from LOY estimates of ovulation suppression based on pregnancies using four different approaches. Subsequent classes of algorithms exclude from LOY estimates ovulation suppression based on oral contraceptive use (second class) and both OC use and breastfeeding (third class). The fourth class of algorithms adjusts LOY based on average cycle length.

			Variables included in LOY algorithms							
Algorithms #	Number of sites included	Sites included	Age at last menstrual period	Age at menarche	Number of pregnancies, regardless of outcome	Total number of months of being pregnant, regardless of outcome(s)	Total number of full-term births	Duration of oral contraceptive use, months	Total months of breastfeeding	LOY algorithms[1]
The first class of algorithms: includes only ovulation suppression due to pregnancy										
A	25	AUS, BAV, CON, DOV, GER, HAW, HOP, JPN, MAY, MCC, NCO, NEC, NJO, NTH, OVA, POL, SON, STA, SWH, TBO, TOR, UCI, UKO, USC, VTL	X	X	X					Menstrual span – number of pregs *0.75
B	22	AUS, CON, DOV, GER, HAW, HOP, JPN, MCC, NCO, NEC, NJO, NTH, OVA, POL, SON, STA, SWH, TBO, TOR, UCI, UKO, USC	X	X			X			Menstrual span – number of full-term births *0.75
C	22	AUS, CON, DOV, GER, HAW, HOP, JPN, MCC, NCO, NEC, NJO, NTH,	X	X	X		X			Menstrual span – number of full-term births *0.75 - (number of pregs – number of full-term births) *0.25

		OVA, POL, SON, STA, SWH, TBO, TOR, UCI, UKO, USC								
D	13	AUS, DOV, GER, HAW, MCC, NCO, NJO, OVA, POL, STA, TBO, UCI, USC	X	X		X				Menstrual span –(months of being pregnant)/12
The second class of algorithms: each of the first-class algorithms with the addition of ovulation suppression due to oral contraceptive use										
E	24	AUS, CON, DOV, GER, HAW, HOP, JPN, MAY, MCC, NCO, NEC, NJO, NTH, OVA, POL, SON, STA, SWH, TBO, TOR, UCI, UKO, USC, VTL	X	X	X			X		Algorithm A – (months of oral contraceptive use)/12
F	22	AUS, CON, DOV, GER, HAW, HOP, JPN, MCC, NCO, NEC, NJO, NTH, OVA, POL, SON, STA, SWH, TBO, TOR, UCI, UKO, USC	X	X			X	X		Algorithm B - (months of oral contraceptive use)/12
G	22	AUS, CON, DOV, GER, HAW, HOP, JPN, MCC, NCO, NEC, NJO, NTH, OVA, POL, SON, STA, SWH, TBO, TOR, UCI, UKO, USC	X	X	X		X	X		Algorithm C 0- (months of oral contraceptive use)/12
H	13	AUS, DOV, GER, HAW, MCC, NCO, NJO, OVA, POL, STA, TBO, UCI, USC	X	X		X		X		Algorithm D - (months of oral contraceptive use)/12
The third class of algorithms: each of the second class of algorithms with the addition of ovulation suppression due to breastfeeding										
I	17	AUS, CON, DOV, GER, HAW, HOP, JPN, NCO, NEC, NJO, NTH, POL, SON, STA, SWH, TOR, USC	X	X	X			X	X	Algorithm E – (months of breastfeeding)/12
J	17	AUS, CON, DOV, GER, HAW, HOP, JPN, NCO, NEC, NJO, NTH, POL, SON, STA, SWH, TOR, USC	X	X			X	X	X	Algorithm F - (months of breastfeeding)/12
K	17	AUS, CON, DOV, GER, HAW, HOP, JPN, NCO, NEC, NJO, NTH, POL, SON, STA, SWH, TOR, USC	X	X	X		X	X	X	Algorithm G - (months of breastfeeding)/12
L	9	AUS, DOV, GER, HAW, NCO, NJO, POL, STA, USC	X	X		X		X	X	Algorithm H - (months of breastfeeding)/12

[1] The menstrual span is defined as age at last menstrual period minus age at menarche

Supplementary Table 2. Comparison of observed and assigned values of age at last menstrual period (LMP). (A). Seven studies in OCAC recorded age at menopause. For observations with an age at menopause < 60, we assigned that age as the age at LMP (average age at LMP was 47.12 years for controls, 47.00 for cases). To assess our imputation algorithm, we imputed age at LMP for these observations using our imputation algorithm (Figure 2). The imputed average age at LMP for these observations was 48.34 for controls and 48.41 for cases. We then applied our imputation algorithm to the observations in those 7 studies that were missing age at menopause or recorded age at menopause as > 60 years old (average age at LMP was 50.58 for controls, 50.22 for cases). Combining the actual observed values when known and imputed data for missing data, the average age at LMP for these 7 studies was 47.60 for controls and 47.42 for cases. When using imputation for all observations in these 7 studies, the average at LMP was 48.71 for cases and 48.70 for controls. **(B)** We then applied our imputation algorithm (Figure 2) to the remaining studies that did not have a recorded age at menopause for any observation. The imputed average age at LMP for all observations in these studies was 48.67 for controls and 48.65 for cases.

		Observations with a known age at menopause < 60 years				Observations with age at menopause missing or >60 years		Combining observations with known age at menopause <60 and imputed values for observations with age at menopause missing or >60		Using imputed values for all observations	
		Using age at menopause as age at LMP		Using imputation algorithm to assign age at LMP		Using imputation algorithm to assign age at LMP					
		Controls	Cases	Controls	Cases	Controls	Cases	Controls	Cases	Controls	Cases
**A.**	N	7162	5974	7162	5974	1154	907	8316	6881	8316	6881
	CON, DOV, HOP, NEC, POL, SON, TOR[a]	47.12 (6.15)	47.00 (6.23)	48.34 (5.77)	48.41 (5.96)	50.58 (2.52)	50.22 (3.68)	47.60 (5.90)	47.42 (6.06)	48.71 (5.54)	48.70 (5.79)
**B**	N					17,888	14,386			17,888	14,386
	All Others Studies[b]	NA	NA	NA	NA	48.67 (5.92)	48.65 (6.13)	NA	NA	48.67 (5.92)	48.65 (6.13)

NA, not applicable.
[a] There are 315 participants without menopause status in the OCAC core dataset.
[b] There are 2004 participants without menopause status in the OCAC core dataset.

Supplementary Table 3. Percentage of missing values in components of lifetime ovulatory years calculation by OCAC site

study	Age at menarche		Hysterectomy		Age at hysterectomy (among women with hysterectomy)		HRT use (no matter what type)		First age using HRT (among women with HRT)		Age at last menstrual period	
	controls	cases	controls	cases	controls	cases	controls	cases	controls	cases	controls	cases
AUS	3.7	7.2	1.1	5.0	1.0	1.8	0	5.2	3.6	16.6	100	100
BAV	46.9	36.7	41.3	55.9	59.1	6.5	48.2	46.8	100	100	100	100
CON[1]	0	0	0	0	3.2	5.4	100	100	-	-	0.7	1.2
DOV[1]	0	0.1	0	0	0	0	0	0	0.1	0	34.2	30.0
GER	1.1	2.7	0	0	0	0	0	0	0	1.9	100	100
HAW	0	0.1	0	0	0	0	0	0	0	0	100	100
HOP	0	0	0	0	0	0	1.9	1.9	0.2	0.4	5.3	4.4
JPN	4.7	0	0	0.7	100	100	2.6	0.7	100	54.6	100	100
MAY	6.6	17.4	100	22.6	100	100	5.2	22.5	11.5	10.4	100	100
MCC	16.1	41.6	4.5	100	-	-	100	100	-	-	100	100
NCO	0.3	0.5	0.1	0.2	1.2	1.23	0.1	0.2	0	0	100	100
NEC	0.4	0.4	0	0	0	0	0	0	6.4	4.5	3.0	2.9
NJO	0.7	0.8	0	100	-	-	0	0	0.7	8.6	100	100
NTH	0.8	14.0	0.5	2.3	3.5	0.6	21.2	1.5	44.0	13.6	100	100
OVA	1.8	2.5	0.1	0.1	2.7	5.0	0	0	3.2	5.7	100	100
POL	1.6	1.0	0	0.3	1.7	0	5.7	6.8	1.9	2.9	1.4	3.4
SON	0	0	0	0	0	0	0	0	1.0	1.2	0.2	0.4
STA	0	2.1	0	0.6	1.6	1.2	0	0.3	0	0	100	100
SWH	0.1	0	0	0	0	0	100	100	-	-	100	100
TBO	41.5	12.3	92.2	46.0	6.7	2.89	100	100	-	-	100	100
TOR	0.3	0.1	0	0.4	0	0	0	0	100	100	0.3	0.5
UCI	7.8	6.8	7.2	6.3	0.6	4.6	11.9	7.7	1.5	0.9	100	100
UKO	11	20.6	11.9	23.4	3.8	5.5	10.4	12.5	4.4	6.5	100	100
USC	0.04	0.1	0	0	0	0	0	0	0	0	100	100
VTL	2.4	3.9	0	0	100	100	100	100	-	-	100	100

Supplementary Table 3 (cont'd)

study	Number of Pregnancies, regardless of outcome		Total number of months of being pregnant, regardless of outcome(s)		Total number of full-term births		Total months of breastfeeding (among parous women)		Duration of Oral Contraceptive Use, months	
	controls	cases	controls	cases	controls	cases	controls	cases	controls	cases
AUS	0	5.2	4.0	22.4	0	4.8	12.4	25.5	0.5	5.8
BAV	45.3	32.6	91.4	88.5	91.4	88.4	57.9	80.6	87.0	87.7
CON[1]	0	0	100	100	0	0	10.5	14.9	0	0
DOV[1]	0.05	0.1	0.6	0.6	0	0.2	10.5	17.0	0	0.2
GER	0	0	0	0	0	0	15.0	20.2	1.1	1.2
HAW	0	0	0	0	0	0	11.3	22.5	0	0
HOP	0	0	90.7	81.6	0	0	9.3	18.5	0	0.1
JPN	2.2	2.0	87.5	80.3	2.1	0.7	16.3	22.4	2.2	2.0
MAY	4.4	10.4	100	100	100	100	100	100	7.2	12.9
MCC	16.4	41.6	16.3	42.2	16.3	42.2	37.2	65.9	18.1	43.9
NCO	0	0.1	0.4	0.5	0	0.1	9.2	16.2	3.2	4.4
NEC	0	0	86.8	75.3	0	0	13.2	24.7	0.05	0.2
NJO	0	0.4	0.4	0.4	0	0.4	10.5	25.3	2.2	3.4
NTH	17.5	1.1	99.7	2.3	16.5	1.1	18.3	25.3	2.3	2.0
OVA	0.1	0.1	27.1	42.3	0.04	0.1	31.5	50.9	0.9	1.1
POL	0	0.3	0.1	0.7	0	0.3	27.6	39.1	1.7	2.0
SON	0	0.2	100	100	0	0	9.7	19.3	0	0
STA	0	0	0	0	0	0	24.0	38.9	0	0.8
SWH	0	0	100	100	0	0	2.3	3.9	0.1	0
TBO	0.5	0.7	49.3	49.1	1.9	2.8	54.1	28.1	44.9	15.4
TOR	0	0	93.2	80.9	0	0	0	0	0	0
UCI	6.7	6.1	6.7	6.1	6.7	6.1	55.0	24.5	7	6.8
UKO	12.2	21.7	91.3	82.7	12.2	21.7	37.4	52.4	16.5	26.7
USC	0	0	0	0.04	0	0	39	39.3	0.2	0.1
VTL	10.5	11.7	100	100	100	100	100	100	1.6	3.9

Supplementary Table 4. Distribution of lifetime ovulatory years calculated from 12 algorithms among participants with complete data

Algorithm	Number of sites included	Controls N (%)	Cases N (%)	mean	standard deviation	median	25th, 75th percentile
A	25	25,081 (55.6)	20,046 (44.4)	33.85	5.98	35.25	32.00, 37.50
B	22	22,519 (55.6)	18,013 (44.4)	34.25	5.91	35.75	32.50, 37.50
C	22	22,509 (55.6)	18,003 (44.4)	34.11	5.93	35.50	32.25, 37.50
D	13	13,617 (56.0)	10,689 (44.0)	34.12	6.21	35.75	32.25, 37.70
E	24	24,480 (55.9)	19,323 (44.1)	30.06	8.31	32.33	26.00, 35.75
F	22	22,316 (55.7)	17,772 (44.3)	30.58	8.18	32.92	26.50, 36.25
G	22	22,306 (55.7)	17,762 (44.3)	30.43	8.19	32.75	26.50, 36.17
H	14	13,515 (56.1)	10,576 (43.9)	30.16	8.45	32.50	25.83, 36.17
I	17	14,900 (56.8)	11,829 (43.2)	29.58	7.95	31.67	25.50, 35.20
J	17	14,902 (56.8)	11,339 (43.2)	30.10	7.88	32.25	26.17, 35.53
K	17	14,900 (56.8)	11,329 (43.2)	29.92	7.89	32.00	26.00, 35.50
L	9	8,473 (56.6)	6,498 (43.4)	29.85	8.13	32.08	25.50, 35.50

Supplementary Table 5. Pairwise correlations of lifetime ovulatory years calculated from 12 algorithms limiting to cases and controls with complete data in each algorithm in the pair-wise comparison

Algorithms	A	B	C	D	E	F	G	H	I	J	K	L
A	1.0000											
B	0.9919	1.0000										
C	0.9964	0.9991	1.0000									
D	0.9791	0.9831	0.9835	1.0000								
E	0.7474	0.7526	0.7556	0.7398	1.0000							
F	0.7488	0.7556	0.7547	0.7556	0.9957	1.0000						
G	0.7529	0.7559	0.7562	0.7566	0.9981	0.9995	1.0000					
H	0.7431	0.7472	0.7472	0.7567	0.9888	0.9996	0.9998	1.0000				
I	0.7607	0.7553	0.7588	0.7540	0.9968	0.9915	0.9944	0.9936	1.0000			
J	0.7469	0.7556	0.7544	0.7506	0.9918	0.9967	0.9962	0.9963	0.9949	1.0000		
K	0.7524	0.7564	0.7567	0.7526	0.9946	0.9961	0.9967	0.9966	0.9978	0.9994	1.0000	
L	0.7466	0.7517	0.7516	0.7522	0.9937	0.9962	0.9965	0.9967	0.9969	0.9996	0.9998	1.0000

Supplementary Table 6. Correlations (rho) between individual components and the corresponding lifetime ovulatory years from 12 algorithms using complete data

Components	A	B	C	D	E	F	G	H	I	J	K	L
Age at last menstrual period	0.9313	0.9399	0.9386	0.9242	0.7193	0.7332	0.7331	0.7226	0.7291	0.7278	0.7290	0.7272
Age at menarche	-0.2502	-0.2533	-0.2532	-0.2296	-0.1511	-0.1519	-0.1521	-0.1416	-0.1599	-0.1608	-0.1607	-0.1351
Number of pregnancies, regardless of outcome	-0.0488	NA	-0.0028	NA	-0.0181	NA	0.0528	NA	-0.0284	NA	-0.0089	NA
Total number of months of being pregnant, regardless of outcome(s)	NA	NA	NA	-0.0944	NA	NA	NA	-0.0165	NA	NA	NA	-0.0404
Total number of full-term births	NA	0.0502	0.0480	NA	NA	0.1001	0.0984	NA	NA	0.0636	0.0660	NA
Total months of breastfeeding	NA	NA	NA	NA	NA	NA	NA	NA	-0.1326	-0.1311	-0.1318	-0.1254
Duration of oral contraceptive use, months	NA	NA	NA	NA	-0.6939	-0.6884	-0.6874	-0.6775	-0.6916	-0.6992	-0.6974	-0.6940

Supplementary Figure 1: Forest Plot from Meta-Analysis of the Association Between Lifetime Ovulatory Years and Epithelial Ovarian Cancer. Using algorithm K, we conducted a meta-analysis and pooled estimate using (**A)** all studies and (**B**) excluding JPN and SWH.

A.

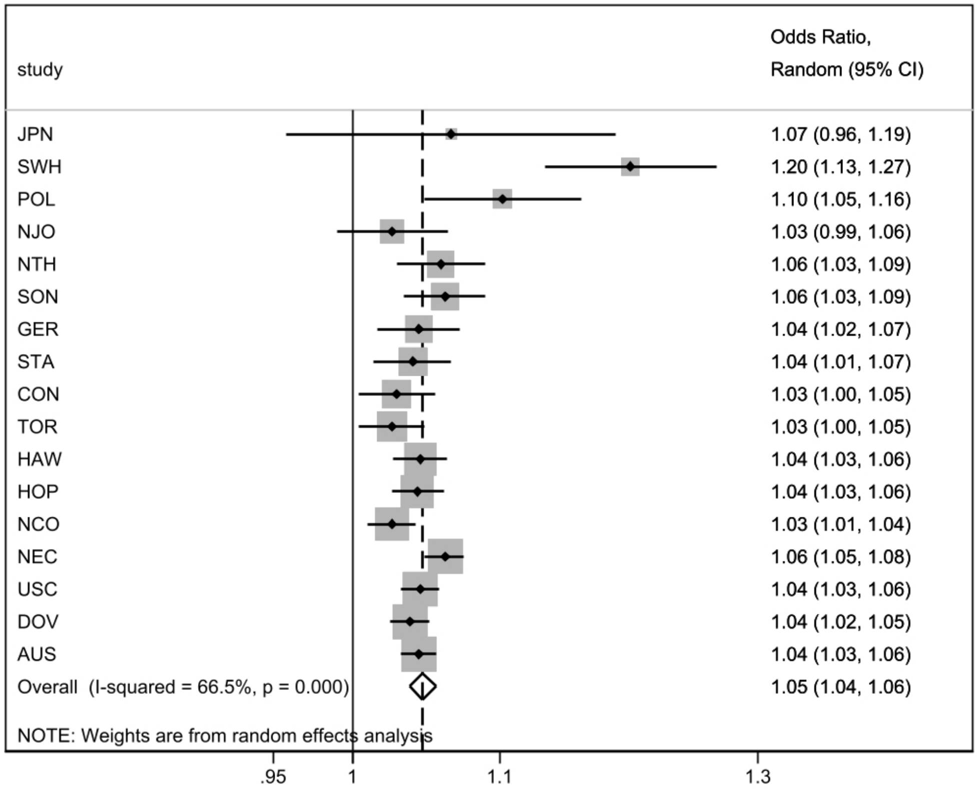

B.

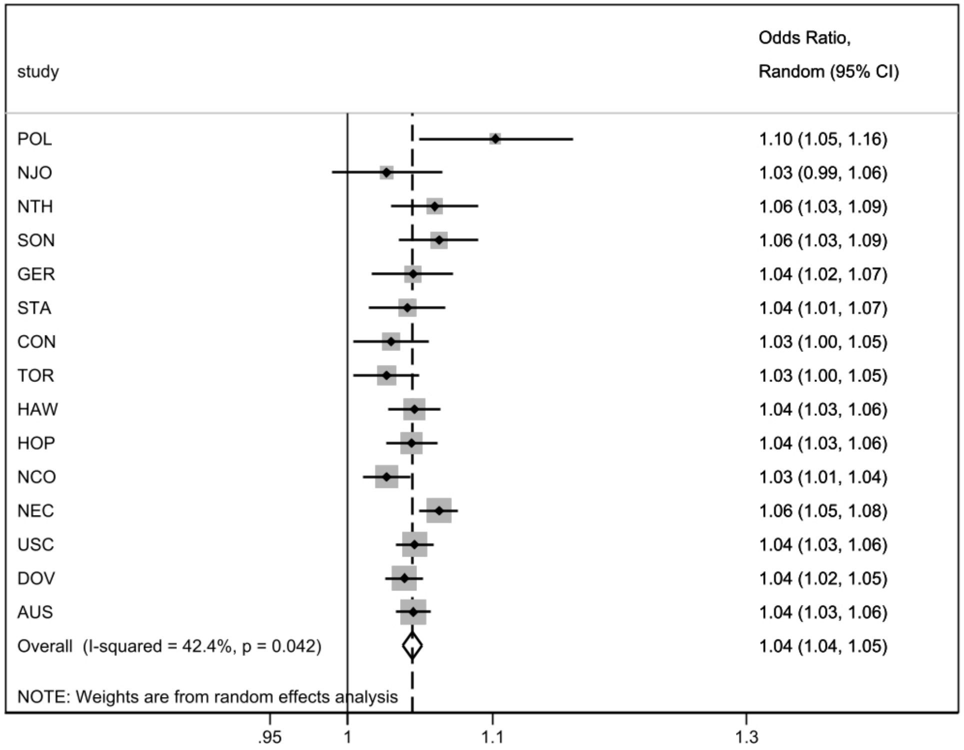
study
Odds Ratio,
Random (95% CI)
POL 1.10 (1.05, 1.16)
NJO 1.03 (0.99, 1.06)
NTH 1.06 (1.03, 1.09)
SON 1.06 (1.03, 1.09)
GER 1.04 (1.02, 1.07)
STA 1.04 (1.01, 1.07)
CON 1.03 (1.00, 1.05)
TOR 1.03 (1.00, 1.05)
HAW 1.04 (1.03, 1.06)
HOP 1.04 (1.03, 1.06)
NCO 1.03 (1.01, 1.04)
NEC 1.06 (1.05, 1.08)
USC 1.04 (1.03, 1.06)
DOV 1.04 (1.02, 1.05)
AUS 1.04 (1.03, 1.06)
Overall (I-squared = 42.4%, p = 0.042) 1.04 (1.04, 1.05)
NOTE: Weights are from random effects analysis
.95
1
1.1
1.3
